# Developing student-centred perspectives in PBL: how teacher profiles reveal educational needs for faculty development programmes

**DOI:** 10.1186/s12909-023-04538-0

**Published:** 2023-08-16

**Authors:** Lukas Daniel Leatemia, Astrid Pratidina Susilo, Jeroen Donkers, Jeroen J. G. van Merrienboer

**Affiliations:** 1 Department of Medical Education, Faculty of Medicine, Mulawarman University, Samarinda, East Kalimantan, Indonesia; 2 Department of Medical Education and Bioethics, Faculty of Medicine, Universitas Surabaya, Surabaya, East Java, Indonesia; 3 Department of Educational Development and Research, Faculty of Health Medicine and Life Sciences, Maastricht University, Maastricht, the Netherlands

**Keywords:** Teacher profiles, Student-centred perspectives, Onion model, PBL, Faculty development

## Abstract

**Background:**

In Asian higher education, PBL is not always successful, as few teachers have embraced a student-centred perspective. To cultivate such essential perspectives, faculty development programmes should address teachers’ specific educational needs, which sadly is currently not sufficiently the case. This study aimed to identify teacher profiles that would reveal these specific educational needs of teachers and to investigate the relationship between these profiles and the amount of PBL training previously received.

**Methods:**

To identify the said profiles, we performed latent profile analysis on a stratified random sample of 543 teachers based on a survey of teaching perspectives on the six aspects of Korthagen’s onion model of reflection (environment, behaviour, competencies, beliefs, identity and mission). Additionally, we employed Chi-square and Mann-Whitney tests to investigate the aforementioned relationship.

**Results:**

We identified six teacher profiles that resemble the diffusion of innovations theory’s classification of innovation adopters: Innovators, Early adopters, Early majority 1, Early majority 2, Late majority and Laggards. The Chi-square test demonstrated that the amount of PBL training received did not differ significantly across profiles, although teachers with a more innovative profile had undergone slightly more PBL training. The Mann-Whitney test furthermore revealed for three profiles that more PBL training was associated with a higher overall score for student-centredness. When aspects were considered separately, however, this was not the case.

**Conclusions:**

The findings confirmed that current faculty development programmes are not sufficiently tailored to teachers’ needs. We therefore propose that faculty development programmes be redesigned to address teachers’ specific educational needs as reflected in the profiles based on the 6 aspects of the onion model. We expect such a tailored approach to more effectively promote the development of student-centred perspectives.

## Background

Two decades after problem-based learning (PBL) was first introduced in Asian higher education, many teachers still have not embraced a student-centred perspective, despite having received a certain amount of PBL training [1]. They continue to adopt a teacher-centred perspective and are resistant to interventions that aim to render their teaching approaches more student-centred [1, 2]. A potential reason is that not all faculty development programmes sufficiently address their specific educational needs. Rather than targeting teachers’ beliefs, professional identity and mission [3, 4], these programmes essentially focus on teachers’ competencies and behaviour (e.g. what to do in a tutor group). The present study, therefore, sought to identify teacher profiles that indicate the specific educational needs of groups of teachers for faculty development purposes. Additionally, it aimed to investigate the relationship between these profiles and the amount of PBL training teachers had previously received.

**Fig. 1:**
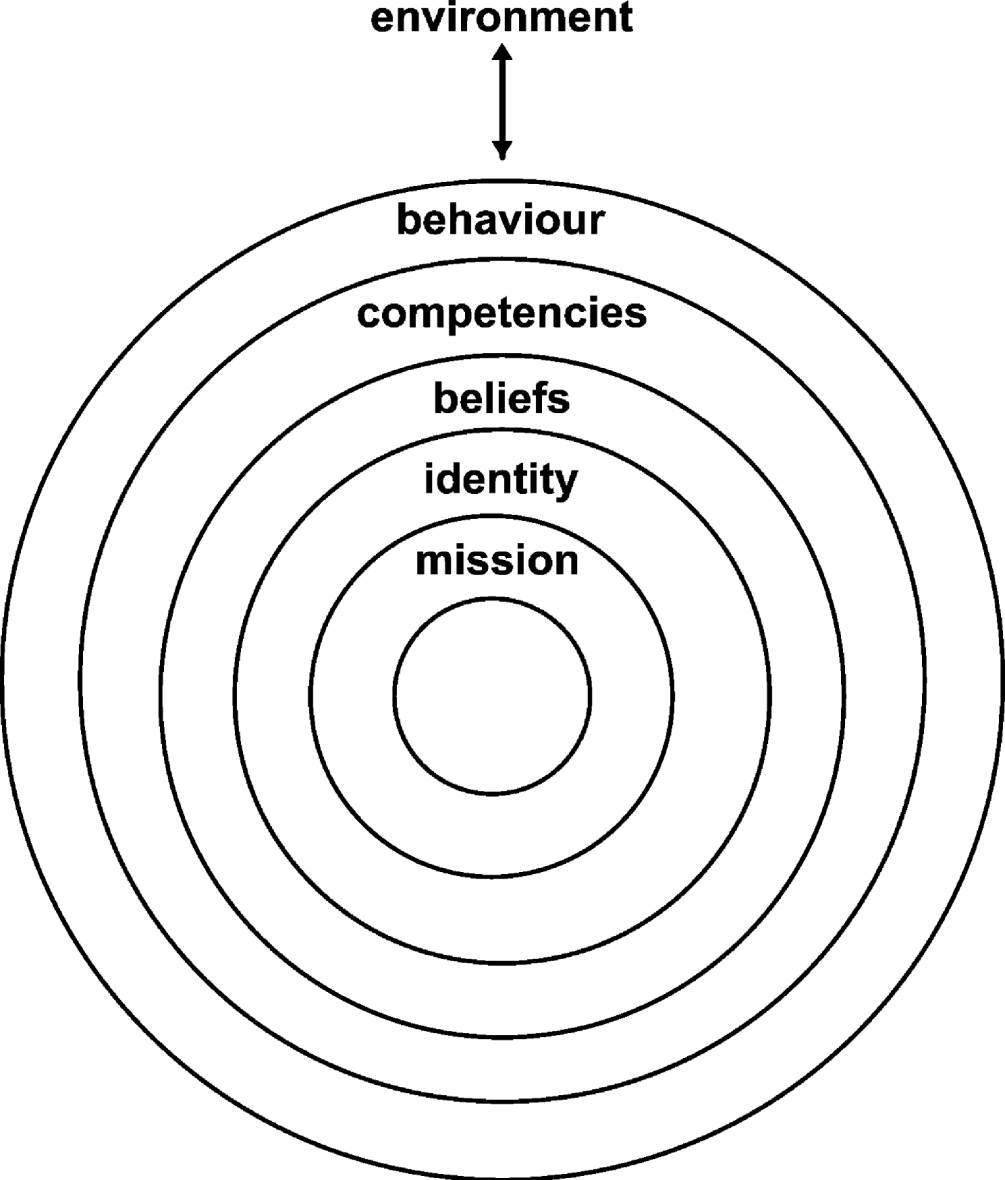
The onion model [5]

In operationalising the said needs of teachers, we drew from Korthagen’s onion model, which is a theoretical framework describing six levels that may influence teacher performance [5]. The framework structure resembles a slice of onion cut in the middle. From the outer to the inner rings, the levels reflect teachers’ environment, behaviour, competencies, beliefs, identity and mission, respectively (see Fig. 1). As such, the structure illustrates how each of the levels may influence the other levels, from the inner to the outer levels, and vice versa. Ideally, all levels should be coherent and in complete alignment. When this is not the case, teachers may experience problems in showing adequate or effective behaviour [5, 6].

The first level, the environment, refers to everything from the outside that teachers may encounter during their teaching activities. On the second level, we find teachers’ behaviour which represents their reactions to the environment or to particular circumstances and their related actions. Third, teachers’ competencies denote their knowledge, skills and attitudes regarding teaching, whereas beliefs (Level 4) refer to the value teachers attach to their students’ teaching and learning. Finally, identity (Level 5) captures how teachers define or see their professional identity, and their mission (Level 6) summarises what they hope to accomplish through their work, or the calling that has led them to become a teacher [5, 6].

PBL is a student-centred constructivist pedagogy using ‘trigger’ problems as a basis for learning to increase knowledge and understanding [7, 8]. Teachers perform a student-centred approach to facilitate students’ learning to be more self-directed, contextual, collaborative, and constructive [7]. In PBL, teachers function more effectively when their perspectives on all the six levels or teaching aspects reflect student-centredness. When it comes to the environmental aspect, for instance, teachers should feel that the institution supports their professional development, facilitates discussions on PBL implementation, provides adequate teaching and learning facilities, evaluates their performance in PBL sessions, and adequately balances rewards with punishment [9, 10]. In terms of behaviour, teachers should feel that they encourage and stimulate constructive/active, self-directed, contextual and collaborative learning during PBL activities among their students [11]. As for competencies, they should consider themselves capable of facilitating tutorial PBL sessions as teachers who stimulate and ask questions, provide information, observe, analyse and provide feedback [12]. With respect to their beliefs, teachers should value active learning by their students as well as student-centred relationships [13]. Finally, teachers should feel that they have didactical and pedagogical expertise (the ‘identity’ aspect), while also being patient, calm, taking pleasure in teaching, and being enthusiastic about their profession (the ‘mission’ aspect) [14].

When one or more of these aspects does not reflect student-centredness, however, teachers may struggle to perform well in PBL. Information about these struggles among groups of teachers may provide valuable input to faculty development programmes that take the needs of those specific groups into account [5, 15]. Teachers in one group might profit from training that focuses on changing their beliefs, whereas other teachers might need to work on their competencies. At the same time, faculty development programmes that target precisely these beliefs and competencies might be useless to yet other teachers who need to learn to deal with, for instance, the environment.

As previously stated, the second focus of this study was to investigate the relationship between the amount of PBL training received and the teacher profiles. We hypothesised that the amount of prior training would differ across profiles (i.e. teachers with more student-centred profiles would have received more PBL training) and that more PBL training would give higher scores for some aspects but not for others. With this exploration, we aimed at gaining more insight into whether current faculty development programmes, such as PBL training, are appropriately tailored to the needs of specific teacher groups.

PBL was an educational innovation for all teachers. Therefore, the diffusion of innovation theory is used to understand how teachers adopt PBL as reflected by their student-centred perspectives in each profile. The diffusion of innovations theory’s classification of innovation adopters are innovators, early adopters, early majority, late majority, and laggards. The early adopter is a group of teachers who first adopt PBL as an innovation with a high level of student-centred perspectives on the inner aspects and fewer boundaries from outer aspects, such as the environment. They are the leaders and role models for other teachers to embrace innovation. Innovators are willing to experience new ideas, although their perspectives on the outer aspects, such as the environment, do not support them. The early majority adopts the innovation after a few other teachers adopt it, while the late majority will adopt it after the majority of teachers adopt it. The laggards relatively have extended time to embrace the innovation because they are more skeptical about it, and their outer aspects do not support the PBL implementation. We identified laggards as a group of teachers with the lowest student-centred perspective [25, 26].

That said, this study complements previous studies that developed teacher profiles based on teachers’ competencies and behaviour, such as student and teacher learning, teaching approaches and teachers’ uncertainty [16–19]. Consequently, faculty development programmes tend to focus mostly on teachers’ competencies and behaviour rather than targeting their environment, beliefs, identity and mission [4, 5]. Therefore, our strategy to identify the needs of specific teacher groups for faculty development purposes is new in that we aimed to construct teacher profiles based on the six aspects of the onion model in a large-scale quantitative approach. To this end, we addressed the following research questions: (1) What teacher profiles can we distinguish based on the six aspects of the onion model? (2) Do teachers in the different profiles differ in the amount of PBL training they have already received? (3) Does the amount of previous PBL training within each profile distinctly influence the degree of student-centredness of each of the onion model’s six aspects?

## Methods and materials

### Setting

We conducted a cross-sectional survey across 20 medical schools in different regions of Indonesia. In 2006, the Indonesian government introduced legislation requiring all medical schools to implement PBL in their curricula. The schools varied in age from 5 to 66 years old. Those that were established before 2006 had transformed their conventional, lecture-based curriculum into a PBL curriculum, whereas those founded after that year had started off with a PBL curriculum.

The schools varied in their implementation of PBL; although they often combined small-group discussions with traditional lectures, the number of lectures per week differed across schools. Lecturing is dominant, around 50–60% of total learning activities. In one week, students usually attended 2–3 small group discussions, which lasted 2 h per session. In the discussion, teachers facilitated students to learn based on one or more problems, helped them to define learning objectives, and refined acquired knowledge. Between the discussions, students engaged in self-study and attended lectures 4–6 times, lasting 2 h per lecture, and one skills training lasting 3 h.

All the schools had their regulations and methods to evaluate and train their tutors. They have a unit/ department of medical education to develop and evaluate teachers’ curricula and training. They obliged their teachers to join formal PBL training conducted by the schools. Such activity typically lasted for one or two days, approximately 6 to 7 h per day. Training contents and methods varied, mostly conducted in the traditional format, such as seminars and workshops to increase teachers’ competencies as tutors.

### Participants

Participants were 543 full-time teachers from 20 medical schools in Indonesia who had been actively involved in tutoring PBL sessions for more than one year. We selected the schools using a stratified random sampling method from the six areas described by the Indonesian Medical Education Association (IMEA). The IMEA groups medical schools in Indonesia into six areas based on their geographical positions, specifically: Sumatera (Area 1), Jakarta (Area 2), West Java (Area 3), Central Java, Jogjakarta and Kalimantan (Area 4), East Java, Bali and West and East Nusa Tenggara (Area 5) and Sulawesi, Maluku and Papua (Area 6).

### Materials

We used the Student-centred Perspective of Teachers (SCPT) questionnaire to measure teachers’ perspectives on the six teaching aspects of the Onion model [20]. This questionnaire is a new instrument, with internal and external validation from our previous study. The validated instrument spans 19 items over six subscales that correspond with the six aspects of the Onion model: environment, behaviour, competency, belief, identity and mission. Confirmatory factor analysis (CFA) revealed that these six subscales were fit after revision with the following Composite Reliability scores: environment (0.72), behaviour (0.74), competencies (0.63), beliefs (0.55), identity (0.76) and mission (0.60). All items were rated on a 5-point Likert scale ranging from 1 (strongly disagree) to 5 (strongly agree). Higher scores indicated higher levels of student-centredness. Moreover, the ANOVA test for the external validation indicated that the amount of PBL training received significantly affected all subscales. Example items of the SCPT questionnaire are listed in Table 1 below.

**Table 1:** Examples of items from the SCPT questionnaire

Subscale	Items
Environment	• My institution facilitates discussion with all lecturers to discuss PBL (views/concepts, small-group discussion processes, etc.) routinely
	• My institution evaluates the implementation of the PBL curriculum periodically through a specific unit/agency
Behaviour	• I encourage students to apply their knowledge to the issues discussed
	• I encourage my students to link their learning goals with the prior knowledge they have
Competency	• I have the ability to stimulate student discussion using formal and informal communication
	• I am able to ask open-ended questions to give students a better understanding of the task
Belief	• Learning in a small-group discussion encourages students to learn
	• Group discussion of a topic will help students to learn about how to get a deep understanding from various points of view
Identity	• I am happy to provide assistance in solving learning problems faced by students
	• It is important for me to help students apply what they have learnt in their daily lives
Mission	• I am open to new ideas and experiences
	• In discussion with my students, I do not feel disturbed by opinions that differ from mine

### Procedure

We used Qualtrics^XM^ to distribute the questionnaire. Participants received the questionnaire through online messages and emails. We invited them to complete the questionnaire, which took approximately 10 min. Before doing so, participants signed an informed consent form and filled in a demographics survey. They also received a reminder three and 10 days after the questionnaire was sent.

### Data analysis

#### Latent Profile Analysis

To identify specific teacher profiles, we used Latent Profile Analysis (LPA). LPA assumes that clusters of individuals have similar ways of responding to a set of certain continuous variables [21]. In this case, we used the responses to the six subscales of the SCPT, assuming that these were continuous, although they were sums of Likert scale items.

For the analysis, we identified the various kinds of model-based clustering and dimension reduction using R with the module *mclust*. First, we translated the responses to the six aspects to a 0–1 scale. To see the distinctions between clusters when the profiles were plotted, all of the indicators required standardisation. We conducted the Bayesian Information Criterion (BIC) plot for all models and then compared the respective outcomes with the Integrated Completed Likelihood (ICL) values to ensure that the chosen solution was the correct one. Finally, we performed the Bootstrap Likelihood Ratio Test (BLRT) to see the suggested numbers of profiles. A *p*-value of ≤ 0.05 indicated more profiles fit better, while *p*-values of > 0.05 indicated less profiles fit better. We used the six profiles’ overall sample means, and conditional response means to interpret each profile substantively and to see how the profiles differed with regard to the aspects [21, 22].

### The differences across teacher profiles in the amount of PBL training received

We used the Chi-square test to explore differences in the amount of PBL training undergone by teachers in the six profiles. The *p*-value was set at 0.05 [23]. We categorised teachers into two groups according to the amount of PBL training previously received, that is, a group who had attended PBL training two times or less (labelled as ‘low’) and a group who had attended PBL training three times or more (labelled as ‘high’).

### The differences within each profile in the amount of PBL training received for the six aspects

When we checked the scores using Kolmogorov-Smirnov and Shapiro-Wilk tests, the data were not distributed normally. We therefore used the Mann-Whitney U test to explore differences in the degree of student-centredness of each separate aspect in relation to the amount of PBL training received. We computed median and interquartile range (IQR) scores for data analysis [23]. Finally, we applied a Bonferroni correction to adjust the *p*-values applicable to the degree of student-centredness per aspect to avoid a type-I error (the false rejection of a null hypothesis) because of the multiple comparison testing [24]. After this correction, the *p*-value was set at 0.0083.

### Ethical considerations

All participants agreed to participate in this study by signing the informed consent form. We ensured confidentiality of participants’ identities in all study reports and any related publications. We obtained ethical approval from the Institutional Ethics Committee of Abdul Wahab Sjahranie Hospital, East Kalimantan, Indonesia (approval no. 179/KEPK-AWS/I/2020).

## Results

Of the 795 teachers we invited, a total of 543 (68.3%) participated in this survey. After a preliminary analysis, we eliminated three participants who had more than five missing values, and imputed the mean score of the items in the respective subscale for 20 participants who had less than five missing values. The remaining sample included 540 participants whose demographic characteristics are listed in Table 2 below.

**Table 2:** Demographic characteristics of the participants

Demographic characteristics	
Age (in years)	From 26 to 72 (M = 41.1, SD = 8.7)
Teachers’ experience (in years)	From 1 to 41 (M = 10.6, SD = 7.3)
Gender	173 male (32%) and 367 female (68%)
Educational background	54 bachelor’s (10%), 393 master’s (72.8%) and 93 doctoral degree (17.2%)
Discipline	190 clinicians (35.2%), 214 basic scientists (39.6%), and 136 others (25.2%)

Note: SD = standard deviation; M = mean; ‘Others’ in the discipline section refers to teachers from other disciplines outside of medical education, such as psychologists and pharmacists

Table 2 shows that the range of the age was wide. However, the distribution of participants’ age was almost similar among the profiles. The median scores are around 38–41 years old, with a wide range of participants’ age among the profile. There were no particular age groups concentrated in a specific profile. It might indicate that the acceptability and motivation levels among faculty across ages were inconsistent with their experience.

### Teacher profiles

A bootstrap likelihood ratio test of the LPA revealed the optimum number of profiles for the EEV and EEE models (see Table 3). The models showed that a six-profile solution fit better than one-, two-, three-, four- and five-profile solutions (*p*-values < 0.05), although it was not statistically different from the seven-profile solution (*p*-value > 0.05). The appropriate choice was a six-profile solution with the EEV model, because the latter was the least restrictive and the likelihood ratio test preferred six profiles.

**Table 3:** BLRT for the EEV and EEE models

Solutions	EEV model (p)	EEE model (p)
2 profiles	81.86 (0.001)	25.01 (0.005)
3 profiles	279.99 (0.001)	192.83 (0.001)
4 profiles	160.33 (0.001)	3.19 (0.748)
5 profiles	413.42 (0.001)	56.92 (0.001)
6 profiles	153.38 (0.001)	365.30 (0.001)
7 profiles	-77.59 (0.754)	-332.67 (0.893)

Note: EEV model = ellipsoidal, equal volume, shape and orientation model; EEE model = ellipsoidal, equal volume and equal shape model

**Fig. 2:**
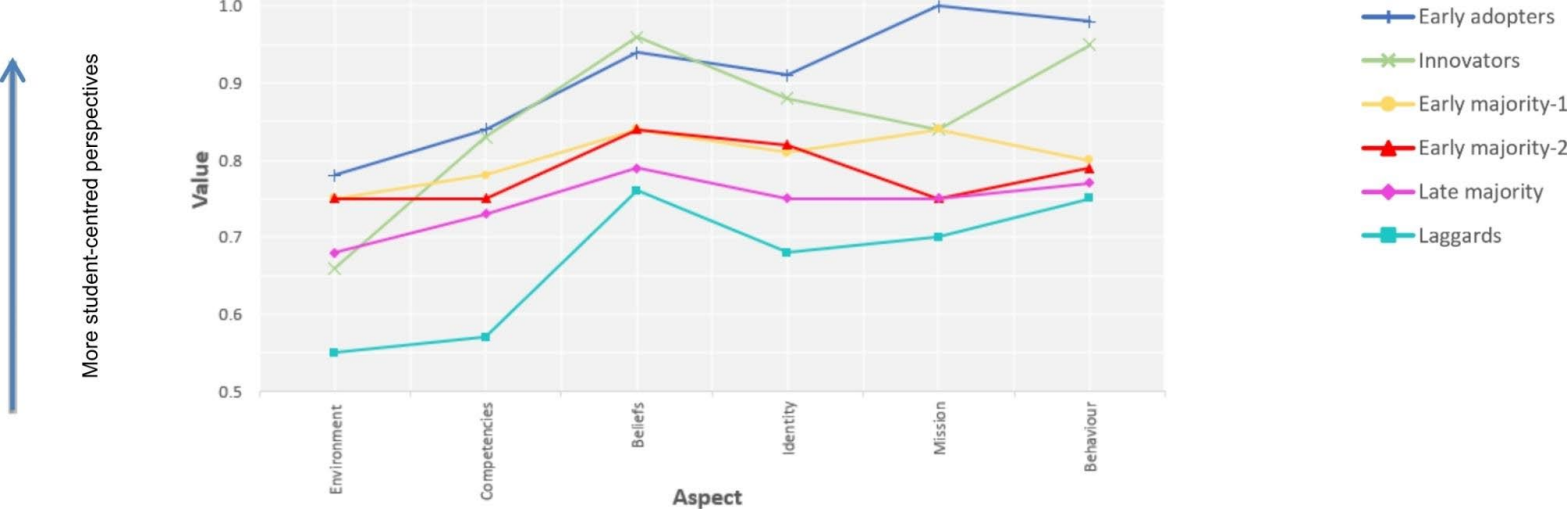
Conditional response means of the six aspects of the onion model relevant to each of the six teacher profiles

Table 4; Fig. 2 present the overall sample means and conditional response means relevant to the six profiles, which we used to interpret each profile substantively. We interpreted the conditional response means of the six aspects of the onion model for each of the six profiles as the positions of the respective group of teachers on the adoption of an innovation (i.e. a PBL curriculum). These positions resemble the diffusion of innovations theory’s classification of innovation adopters, specifically: Innovators, Early adopters, Early majority, Late majority and Laggards [23, 24]. The third profile (Early majority) was split in two – Early majority 1 and Early majority 2 – because despite their differences, they shared many characteristics in common.

**Table 4:** Overall sample means (SD) and conditional response means (SD) of the six aspects of the onion model relevant to each of the six teacher profiles

Profile	n (%)	Environment	Behaviour	Competencies	Beliefs	Identity	Mission
Profile 1 (Early adopters)	121 (22.4)	0.78 (0.20)	0.98 (0.09)	0.84 (0.12)	0.94 (0.09)	0.91 (0.09)	1.00 (0.00)
Profile 2 (Innovators)	49 (9.1)	0.66 (0.26)	0.95 (0.06)	0.83 (0.12)	0.96 (0.06)	0.88 (0.09)	0.84 (0.08)
Profile 3 (Early majority 1)	60 (11.1)	0.75 (0.18)	0.80 (0.09)	0.78 (0.56)	0.84 (0.12)	0.81 (0.10)	0.84 (0.06)
Profile 4 (Early majority 2)	40 (7.4)	0.75 (0.17)	0.79 (0.14)	0.75 (0.15)	0.84 (0.13)	0.82 (0.10)	0.75 (0.18)
Profile 5 (Late majority)	253 (46.8)	0.68 (0.16)	0.77 (0.06)	0.73 (0.07)	0.79 (0.09)	0.75 (0.07)	0.75 (0.00)
Profile 6 (Laggards)	17 (3.2)	0.55 (0.23)	0.75 (0.06)	0.57 (0.15)	0.76 (0.05)	0.68 (0.07)	0.70 (0.11)

The first Profile (Early adopters) comprised 22.4% of the teachers (*N* = 121). Teachers with this profile had a highly student-centred take on beliefs, identity, mission and behaviour, but only a moderately student-centred view on the environment and competencies. The mission aspect received the highest score. As these teachers encountered few environmental obstacles in adopting an innovation, they were labelled ‘*Early adopters’*.

The second profile (Innovators) comprised 9.1% of the teachers (*N* = 49) and represented teachers who had a highly student-centred perspective on behaviour, beliefs and identity, an intermediary student-centred view on competencies and mission, whilst their perceptions of the environment showed few signs of student-centredness. These teachers were coined ‘*Innovators*’ because their highly student-centred take on inner aspects led them to adopt innovation with few obstacles from their competencies and mission, although they did feel constrained by their environment.

Profiles 3 and 4 (Early majority 1 and 2) comprised 11.1% (*N* = 60) and 7.4% (*N* = 40) of the teachers, respectively. Although the two groups shared several characteristics in common, Profile 4 had a lower score for mission. Both groups had intermediary student-centred perspectives on all aspects. They were labelled as ‘*Early majority’* because teachers adopted the innovation if their environment (i.e. leaders and other teachers) supported it.

Referred to as ‘*Late majority*’, Profile 5 included 46.8% (*N* = 253) and hence the majority of teachers. These teachers had intermediary student-centred perspectives on beliefs, identity, mission and behaviour, and perceived the environment and competencies with relatively little student-centredness. As such, they were a little resistant to change and seemed to adopt the innovation only when adopted by their environment as well.

The last profile (Laggards) comprised only 3.2% of the teachers (*N* = 17). The teachers with this profile exhibited a low degree of student-centredness vis-à-vis their environment, competencies, identity and mission, and intermediate levels with regard to their beliefs and behaviour. The environment received the lowest scores, followed by competencies. We flagged these teachers as ‘*Laggards*’ because they were reluctant to change their teacher-centred perspectives, and were sceptic about their environment.

### Differences in the amount of PBL training received across teacher profiles

Table 5 presents the numbers and percentages of teachers in each profile who had previously received two or less times a PBL training as opposed to those with three or more times a PBL training (‘low’ vs ‘high’). The Chi-square test demonstrated that the distribution between these ‘low’ and ‘high’ groups was not significantly different across profiles (*p* = .071). Nevertheless, there was a tendency among the teachers in the early-adopters (46.3%) and early-majority 2 (50%) groups to have received a fair amount of prior PBL training.

**Table 5:** Differences across teacher profiles in the amount of PBL training previously received

Amount of PBL training previously received	Teacher profiles (N = 540)												p-value*
	Early adopters (n = 121)		Innovators (n = 49)		Early majority 1 (n = 60)		Early majority 2 (n = 40)		Late majority (n = 253)		Laggards (n = 17)		
	n	%	n	%	n	%	n	%	n	%	n	%	
Low (≤ 2 times)	65	53.7	29	59.2	40	66.7	20	50	171	67.6	10	58.8	0.071
High (≥ 3 times)	56	46.3	20	40.8	20	33.3	20	50	82	32.4	7	41.2	

Note: Low = participants had received PBL training two times or less; High = participants had received PBL training three times or more. * *P*-values of ≤ 0.05 were considered significant

### Differences in the amount of PBL training received for the six aspects within each profile

We ran a Mann-Whitney U test to compare the differences between teachers who had received low versus high amounts of prior PBL training in their scores for the six different aspects of the onion model as well as all aspects together. From Table 6 we may infer that the early adopters and innovators (i.e. the more innovative profiles) who had received more PBL training had higher levels of student-centredness for all six aspects combined. Although the respective scores were almost similar for the late majority profile, the *p*-value was significant (*p* = .004). After Bonferroni adjustment, however, scores for most or all aspects in all profiles were not significantly different between the two groups (*p*-values > 0.0083), except for beliefs in the early-adopters and environment and behaviour in the late-majority profiles. We conclude that, for several profiles, more PBL training was associated with a higher score for student-centredness when the six aspects were combined, but not when considered separately.

**Table 6:** Differences in the mean scores for the six aspects according to amount of PBL training received

The aspects		Median (interquartile range) Amount of PBL training (N = 540)		Z score	*p*-value
		Low (≤ 2 times)	High (≥ 3 times)		
**Early adopters (n = 121)**		**(n = 65)**	**(n = 56)**		
Environment		4.00 (1.00)	4.50 (1.00)	-1.96	0.050
Behaviour		4.75 (0.75)	4.75 (0.50)	-0.92	0.360
Competencies		4.33 (0.67)	4.50 (0.92)	-1.84	0.065
Beliefs		5.00 (0.50)	5.00 (0.38)	-2.94	0.003**
Identity		4.67 (0.42)	4.83 (0.50)	-1.71	0.087
Mission		5.00 (0.00)	5.00 (0.00)	0.00	1.00
Over all six aspects		4.47 (0.45)	4.66 (0.34)	-2.88	0.004*
**Innovators (n = 49)**		**(n = 29)**	**(n = 20)**		
Environment		3.50 (2.00)	4.00 (0.88)	-1.45	0.146
Behaviour		4.75 (0.50)	5.00 (0.25)	-1.62	0.105
Competencies		4.33 (0.67)	4.33 (1.00)	-1.03	0.301
Beliefs		5.00 (0.50)	5.00 (0.50)	-0.04	0.970
Identity		4.33 (0.50)	4.83 (0.50)	-2.41	0.016
Mission		4.50 (0.00)	4.50 (0.00)	-0.56	0.577
Over all six aspects		4.28 (0.42)	4.52 (0.29)	-2.13	0.033*
**Early majority 1 (n = 60)**		**(n = 40)**	**(n = 20)**		
Environment		4.00 (1.00)	4.00 (0.50)	-0.99	0.323
Behaviour		4.25 (0.50)	4.25 (0.50)	-0.63	0.527
Competencies		4.00 (0.33)	4.00 (0.33)	-0.50	0.615
Beliefs		4.50 (1.00)	4.50 (0.88)	-0.24	0.810
Identity		4.33 (0.67)	4.25 (0.63)	-0.46	0.647
Mission		4.50 (0.00)	4.50 (0.00)	-0.02	0.987
Over all six aspects		4.26 (0.38)	4.31 (0.33)	-0.14	0.888
**Early majority 2 (n = 40)**		**(n = 20)**	**(n = 20)**		
Environment		4.00 (1.00)	4.00 (0.50)	-1.20	0.232
Behaviour		4.12 (0.44)	4.25 (0.50)	-0.85	0.394
Competencies		4.00 (1.00)	4.00 (0.58)	-1.31	0.190
Beliefs		4.25 (0.88)	4.50 (0.88)	-0.47	0.640
Identity		4.33 (0.63)	4.33 (0.46)	-0.63	0.528
Mission		4.50 (1.50)	4.50 (1.00)	-0.18	0.858
Over all six aspects		4.13 (0.79)	4.14 (0.38)	-0.88	0.379
**Late majority (n = 253)**		**(n = 171)**	**(n = 82)**		
Environment		4.00 (0.50)	4.00 (1.00)	-3.81	0.000**
Behaviour		4.00 (0.25)	4.00 (0.25)	-2.74	0.006**
Competencies		4.00 (0.33)	4.00 (0.00)	-1.70	0.089
Beliefs		4.00 (0.50)	4.00 (0.50)	-0.88	0.381
Identity		4.00 (0.33)	4.00 (0.20)	-0.53	0.594
Mission		4.00 (0.00)	4.00 (0.00)	-2.51	0.012
Over all six aspects		4.00 (0.21)	4.00 (0.24)	-2.85	0.004*
**Laggards (n = 17)**		**(n = 10)**	**(n = 7)**		
Environment		3.00 (1.63)	3.50 (1.00)	-0.90	0.367
Behaviour		4.00 (0.13)	4.00 (0.50)	-0.42	0.677
Competencies		3.17 (1.08)	3.33 (0.67)	-0.30	0.766
Beliefs		4.00 (0.13)	4.00 (0.00)	-1.62	0.106
Identity		3.67 (0.38)	3.83 (0.50)	-1.35	0.178
Mission		3.75 (0.50)	3.50 (1.00)	-0.31	0.753
Over all six aspects		3.66 (0.20)	3.71 (0.15)	-0.34	0.732

Note: low = participants had received PBL training two times or less; high = participants had received PBL training three times or more. *P-values were significant if ≤ 0.05. **P-values were significant if ≤ 0.0083

## Discussion

The present study sought to identify teacher profiles based on teachers’ perspectives on the six teaching aspects of the onion model. The analysis revealed six teacher profiles that reflected teachers’ different needs as judged from their low student-centredness scores for the six aspects. We also found that the amount of PBL training teachers had previously received was not significantly different across profiles, although for three profiles more PBL training was significantly associated with higher overall student-centredness, but not when aspects were considered separately.

To answer our first research question (What teacher profiles can we distinguish based on the six aspects of the onion model?), we identified the following six teacher profiles based on the Diffusion of Innovation theory [25, 26]: Early adopters, Innovators, Early majority 1, Early majority 2, Late majority and Laggards. All of the profiles showed opportunities for improvement in terms of their student-centred approach to the aspects, although scores differed across profiles. The teachers with an early-adopter profile, for instance, lacked student-centredness in how they perceived their environment and competencies, while teachers with an innovator profile had low scores for the environment, competencies and mission aspects. Teachers in the early-majority, late-majority and laggard profiles had low scores for all aspects.

For teachers to function effectively in PBL, they should ideally have highly student-centred views on all aspects of the onion model [5, 15]. When this is not the case, they may struggle to embrace a student-centred approach, which is precisely why faculty development programmes should be tailored to their specific educational needs. In this way, these programmes might help teachers to adopt a more student-centred view on all aspects [4, 5, 15].

As for the second research question, we could not confirm that teachers in the various profiles differed in the amount of PBL training they had already received. However, there was a tendency among teachers in the early-adopter and early-majority-2 profiles to have received relatively more prior PBL training. Seeing as teachers’ needs are so diverse, we would not advocate for universal faculty development programmes; rather, to prevent teachers from becoming demotivated and to offer them more precise directions for improving their performance, tailor-made programmes seem indicated [27].

Finally, to answer our third research question about how differences within each profile in the amount of PBL training previously received relate to each of the six aspects, we found that more PBL training was significantly associated with higher overall student-centredness in early adopters, innovators (the more innovative profiles) and the late-majority profile. The same, however, could not be said when aspects were considered separately. These findings suggest that current PBL training is not appropriately tailored to the educational needs of teachers with a specific profile. This resonates with Samarasekera et al. (2020) who argued that, in recent years, Asian higher education tends to organise their faculty development programmes without considering teachers’ educational needs, especially the ‘inner’ aspects. Moreover, these programmes seemingly focus on main outcomes of teacher performance (i.e. their competencies and behaviour), and on skill acquisition in particular. As such, these programmes may only benefit those teachers who need to change their behaviour, while other teachers who might need to work on their ‘inner’ student-centred aspects will experience no gains [5, 15, 27]. By recognising the profiles, faculty development programs could be redesigned to address the specific educational needs of teachers, as reflected in the profiles. We expect such a tailored approach to be more effective in promoting the development of student-centred perspectives of teachers [15].

The reported findings suggest that we should redesign faculty development programmes by tailoring them to the specific educational needs of teachers as reflected in the profiles. Indeed, in proposing his *Professional development 3.0* approach, Korthagen [15] called for a consideration of the unconscious, multi-dimensional and multi-level nature of teachers’ learning by faculty development programmes. ‘Unconscious learning’ implies that programmes should stimulate in-depth reflection to help teachers become more aware of their personal needs and to connect with the theory and practice of PBL. ‘Multi-dimensional learning’ refers to teachers’ cognitive, affective and motivational learning needs that are interfaced with and rooted in the social context, such as series of workshops and seminars [28], longitudinal programmes [29, 30], and workplace learning in community practice [27, 31]. Finally, multi-level learning refers to the six aspects of the onion model that play an important role in influencing teachers’ performance [5, 15]. Combining a variety of teachers’ needs as reflected in the profiles and the other features of teachers learning (i.e. unconscious and multi-dimensional learning) in faculty development programs is an effective approach to supporting teachers to develop their student-centred perspectives [15].

This study has certain limitations. First, the data in this quantitative study is merely taken from teachers’ teaching perspectives through a questionnaire. Although we attempted to use theoretical and researchers triangulation, there was a possibility of the subjectivity of the data, including socially desirable answers to the items in the questionnaire. In future research, it is necessary to confront their teaching perspectives with real situations in their teaching practices (e.g., classroom observations by trained observers) to yield more objective measures. Second, in our survey, we only asked teachers about the amount of PBL training they had received but not about other factors that influence the effectiveness of the training, such as the methods, contents and duration of the training. It is necessary to explore those factors used in existing faculty development programmes to obtain a more comprehensive understanding and teachers’ perceptions of their effectiveness. This study also did not investigate the reason behind the variability among faculty concerning the profiles. Further study is necessary to explore the situations of teachers that encourage or hinder change toward a student-centred perspective based on the six aspects of the Onion model. We, therefore, welcome a qualitative study with in-depth interviews or focus-group discussions with different groups of teachers. Third, the results are limited to the Indonesian context. Therefore, to make these findings more generalisable, teacher profiles to target teachers’ educational needs from other Asian countries with different environments should be identified.

## Conclusion

We identified six teacher profiles that revealed specific needs of groups of teachers for faculty development programmes to address. The amount of PBL training teachers had previously received, however, did not differ significantly across profiles, whilst more PBL training was significantly associated with higher overall student-centredness for three profiles (i.e. early adopters, innovators and late majority), but not when aspects were considered separately. These findings reveal that current faculty development programmes are not sufficiently tailored to teachers’ needs. The said programmes must therefore be redesigned to address the specific educational needs of teachers, as reflected in the profiles based on the six aspects of the onion model. We expect such a tailored approach to be more effective in promoting the development of student-centred perspectives.

## Data Availability

The datasets used during the current study are available from the corresponding author on reasonable request.

## Abbreviations

PBL: Problem Based Learning
SCPT: Student-Centred Perspective of Teachers
LPA: Latent Profile Analysis
ANOVA: Analysis of variance
IMEA: Indonesian Medical Education Association
SCPT: Student-centred Perspective of Teachers
CFA: Confirmatory factor analysis
BIC: Bayesian Information Criterion
ICL: Integrated Completed Likelihood
BLRT: Bootstrap Likelihood Ratio Test
IQR: interquartile range
EEV: ellipsoidal, equal volume
EEE: ellipsoidal, equal volume and equal shape
